# Chronic Suppressive Antibiotic Treatment for Staphylococcal Bone and Joint Implant–Related Infections

**DOI:** 10.3390/antibiotics12050937

**Published:** 2023-05-21

**Authors:** Giancarlo Ceccarelli, Beatrice Perciballi, Alessandro Russo, Paolo Martini, Francesco Marchetti, Marco Rivano Capparuccia, Giancarlo Iaiani, Silvia Fabris, Massimo Ciccozzi, Ciro Villani, Mario Venditti, Gabriella D’Ettorre, Daniele De Meo

**Affiliations:** 1 Department of Internal Medicine, Endocrine-Metabolic Sciences and Infectious Diseases, Policlinico Umberto I University Hospital, 00161 Rome, Italy; 2 Department of Public Health an Infectious Diseases, Sapienza University of Rome, 00161 Rome, Italy; 3 M.I.T.O. (Infections in Traumatology and Orthopedics Surgery) Study Group, Policlinico Umberto I University Hospital, 00161 Rome, Italy; 4 Department of Anatomical, Histological, Forensic Medicine and Musculoskeletal System Sciences, Sapienza University of Rome, 00161 Rome, Italy; 5 Infectious and Tropical Disease Unit, Department of Medical and Surgical Sciences, “Magna Graecia” University of Catanzaro, 88100 Catanzaro, Italy; 6 Plastic Surgery Outpatient Clinic, Villa Mafalda Hospital, 00199 Rome, Italy; 7 National Center for Control and Emergency Against Animal Diseases and Central Crisis Unit, Office III, Directorate General for Animal Health and Veterinary Drugs, Italian Ministry of Health, 00153 Rome, Italy; 8 Unit of Medical Statistics and Molecular Epidemiology, University Campus Bio-Medico of Rome, 00128 Rome, Italy

**Keywords:** periprosthetic joint infection, fracture-related infection, osteomyelitis, debridement, leukocyte scintigraphy, minocycline, suppressive therapy, bone infection

## Abstract

Prosthetic joint infection (PJI) and fracture-related infection (FRI) are difficult-to-treat conditions in patients with severe comorbidity or significant surgical risk. In cases not eligible for standard strategy, debridement procedures with the retention of prosthesis or internal fixation device, combined with long-term antibiotic treatment and subsequent indefinite chronic oral antimicrobial suppression (COAS), can be the only reasonable choice. The aim of this study was to investigate the role of COAS and its follow-up in the management of these cases. We retrospectively analyzed a cohort of 16 patients with a follow-up of at least 6 months (mean age 75 yo, 9F, 7M, 11 PJI, 5 FRI). All microbiological isolates were tetracycline-susceptible staphylococci and for this reason a minocycline-based COAS was adopted after debridement and 3 months of antibiogram-guided antibiotic treatment. Patient monitoring was carried out on a clinical basis, with bimonthly execution of the inflammation indices and serial radiolabeled leukocyte scintigraphy (LS). The overall median time of COAS follow-up was 15 months (min 6–max 30). Moreover, 62.5% of patients were still taking COAS with no relapse after cure at the last evaluation available. Clinical failure with a relapse of the infection was observed in 37.5% of patients; interestingly, 50% of them had previously stopped COAS due to side effects of the antibiotic used. In the COAS follow-up, a combination of clinical, laboratory and LS evaluation seems to monitor the infection properly. COAS can be considered as an interesting approach in patients not suitable for standard treatments of PJI or FRI but it requires careful monitoring.

## 1. Introduction

Periprosthetic joint infection (PJI) and fracture-related infection (FRI) currently remain difficult-to-treat conditions since the course of antibiotic treatment is long, often requiring multiple surgical procedures and prolonged hospitalizations. Moreover, the definitive eradication of causative pathogens can be challenging, and many infections may relapse over time, with a significant negative impact on patient morbidity and health care systems [1,2].

In PJI and FRI, the curative strategy generally includes the removal or replacement of the prosthesis or internal fixation device combined with medical treatment [3]. Nevertheless, in patients with high co-morbidity or significant surgical risk that cannot afford multiple surgery procedures, and in some acute cases (if the implant is stable), debridement procedures (with the retention of prosthesis or internal fixation device) combined with long-term antibiotic treatment are the only reasonable choice [4]. In these cases, after the debridement and a long-term antibiotic treatment course, a subsequent indefinite chronic oral antimicrobial suppression (COAS) can be a choice when all other solutions are not feasible [5,6,7]. Currently, a univocal management and monitoring strategy has not yet been identified in this setting despite the relevance of the topic.

In addition to classic monitoring methods, such as inflammation indices and clinical evaluation, radiolabeled leukocyte scintigraphy (LS) has been proposed. LS is a diagnostic method now suggested in the setting of PJI by the recent guideline released by the European Association of Nuclear Medicine (EANM), endorsed by the European Society of Clinical Microbiology and Infectious Diseases (ESCMID) [8], and also included as a likely criterion in the EBJIS definition of PJI [9]. In fact, LS is useful in the diagnosis of infections, especially in hip and knee PJI, with high rates of sensitivity and specificity (up to 90% according to some authors), and may also provide valuable information in the follow-up of these infections [10,11].

Furthermore, LS has made significant contributions to discriminating soft tissue infection from osteomyelitis and improved diagnosis in terms of localization and the extent of disease [12]. The correct follow-up of treated patients is not yet standardized and remains a challenge for clinicians.

The aim of this study was to focus on the efficacy of COAS in patients with chronic PJI and FRI ineligible for standard treatment in a real-world setting. Moreover, we investigate the strategies for monitoring the efficacy of COAS and the possible role of LS in the chronic management of these cases.

## 2. Materials and Methods

*Design of the study and population*: This is a retrospective analysis of a cohort of patients affected by PJI or FRI not treated with standard strategies due to their high co-morbidity burden or significant surgical risk or surgical/anesthetic contraindications. Debridement procedures with a retention of prosthesis or internal fixation device, combined with long-term antibiotic treatment and subsequent indefinite COAS, were adopted in the management as the only reasonable choice.

**Figure 1:**
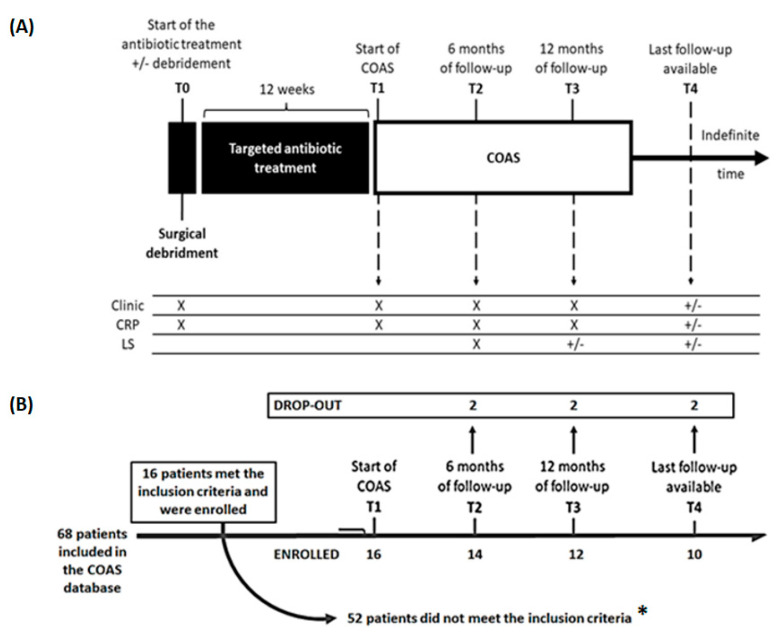
Schematic picture of (**A**) the management of patients and data collection and (**B**) flow-chart of the study. Legend—Clinic: clinical picture; CRP: C reactive protein; LS: Radiolabeled Leukocyte scintigraphy; COAS: chronic oral antimicrobial suppression; (*) Reasons why patients did not meet the inclusion criteria: 34 patients with available follow-up of less than 6 months or lost to follow-up, 18 patients with microbiological isolation other than *Staphylococci*, or with sterile or non-performed microbiological examination.

T0 was considered the start of the antibiotic treatment with or without contextual debridement. T1 was the time of start of COAS, and T2 and T3 were, respectively, 6 months and 1 year of follow-up. T4 was the last follow-up available at least after 1 year of COAS (Figure 1A).

*Eligibility*: From this cohort of patients, we considered eligible subjects with the following characteristics: (1) age >18 years old, (2) cases not eligible for curative surgical treatment according to PJI, (3) a minimum clinical follow-up of 6 months, and (4) microbiological isolates of *Staphylococcus aureus* or coagulase-negative staphylococci obtained in diagnostic or debridement procedures (with at least two microbiological isolates). All infections were defined as chronic (≥4 weeks after surgery if postoperative or ≥3 weeks of duration of the symptoms with hematogenous etiology [3]) and retrospectively confirmed according to EBJIS criteria for PJI and FRI [9,13]. All patients meeting these characteristics were retrospectively recruited from the database of the university referral center for prosthesis infections.

*Definitions*: COAS was defined as a strategy based on a “suppressive antibiotic treatment” in which the administration of antibiotics occurs in the long term or indefinitely over time with the aim of reducing symptoms and delaying or preventing the progression of PJI in cases not eligible for standard surgical treatment [14]. Limited to COAS monitoring, a positive objectivity for one or more of these signs was considered indicative of new exacerbation and failure of COAS: severe joint pain, warmth, redness, tenderness, effusion, restricted active and passive motion, and presence of new fistula or local dehiscence or decubitus. Fever and signs of sepsis were considered indicators of possible systemic spread through bacteremia. Additionally, a new positive result of the LS was considered a sign of failure. The “clinical improvement” was defined as the significant reduction of clinical acute signs of infection present before treatment. The complete clinical disappearance of previous acute signs of infection was defined as “cure”.

Basal evaluation and clinical follow-up: Data for basal evaluation and follow-up monitoring were extracted from the files of patients. Clinical evaluation, laboratory values, and radiological data were reported. Bimonthly execution of the inflammation indices, i.e., C reactive protein (CRP), from the start of treatment were monitored. CRP reference value was <0.5 mg/dL. Moreover, red and white blood cell count and renal and hepatic functions were reported.

*LS*: The results of one or more LS performed in the course of COAS were reported in the dataset. The leukocytes were labeled with Tc-99m- HMPAO using standard methods [15]. A sample of labeled leukocytes was submitted to trypan-blue test to assess the viability of the labeled cells, which was also confirmed by lung, liver and spleen evaluation after 20 min of injection. The mean injected activity was 340 MBq (range: 280–450). Scintigraphy studies were performed using a large-field-of-view digital gamma camera (GE Millennium) equipped with a low energy all-purpose collimator. Static acquisitions of involved bone segment were performed 20 min, 1 h and 24 h after radio-labeled cell re-injection. Static acquisitions were performed in almost two different views, depending on the involved bone segment. Images from each patient were compared by operators during follow-up.

*COAS:* Oral antibiotic agents active against microbiological isolates were chronically used. Side effects and self-reported adherence (assessed by interview during the medical examination) registered on patient files were added to the study database.

*Ethics*: Ethical approval for this study was in accordance with the ethical standards in the 1964 Declaration of Helsinki and US Health Insurance Portability and Accountability Act (HIPAA), and furthermore, informed consent was obtained from all the patients. Ethic Committee approval number was 612/13

*Statistical analysis*: Continuous variables were presented as mean (SD) and categorical variables were expressed as absolute frequencies and percentages. Differences between groups were evaluated by a qualitative analysis by visual inspection and bivariate frequencies comparison and, when appropriate, by Student’s *t*-test, Fisher’s exact test or Chi-Square test, considering α = 0.05.

## 3. Results

From a cohort of 68 patients treated with COAS included in the hospital database, 16 patients met the inclusion criteria and were enrolled in the present study (Figure 1B). Demographic, clinical, and microbiological characteristics at T0 are reported in Table 1.

*Microbiological data*: As per protocol, only cases with Staphylococcus spp isolated and identified from clinical samples of articular fluid or debridement surgical procedures were considered. All S. aureus (2 patients) and coagulase-negative Staphylococci (14 patients) isolates displayed phenotypic oxacillin resistance.

*Surgical debridement and antibiotic treatment*: In all cases, the treatment was started as a consequence of a clinical exacerbation of the chronic infection. All patients were treated with surgical debridement, prolonged targeted antibiotic therapy (12 weeks) and subsequent COAS. Effective antibiogram-guided combined antibiotic treatments were prescribed: an outpatient parenteral antimicrobial therapy (OPAT) based on teicoplanin i.v. (a loading dose of 12 mg/kg q12h for 3–5 doses and then a maintenance dose of 12 mg/kg q24h) for the first 2 months, which was switched to oral antimicrobial treatment, usually with Linezolid (600 mg, 1 tablet q12h). OPAT was eventually associated with one or two other active oral anti-staphylococcal drugs. In all cases, teicoplanin MIC for CONS was <4 mg/L and for *S. aureus* < 2 mg/L. After the debridement procedure plus a 12-week course of targeted antibiotic treatment, significant clinical improvement compared to the start of treatment was reported in 10/16 (62.50%) patients and a cure was reported in 6/16 (37.50%). At the same time, CRP was normal only in 5/16 (31.25%) cases, but in the other 9/16 (56.25%) cases it was increased no more than two times compared to the normal value.

**Figure 2:**
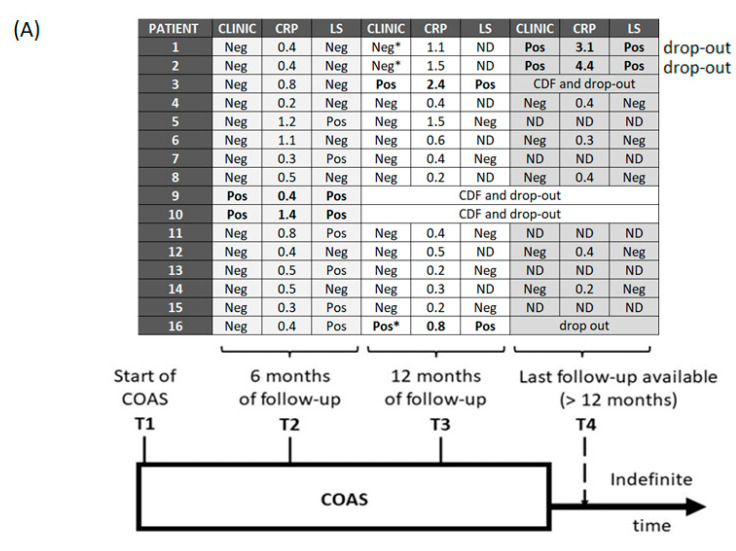

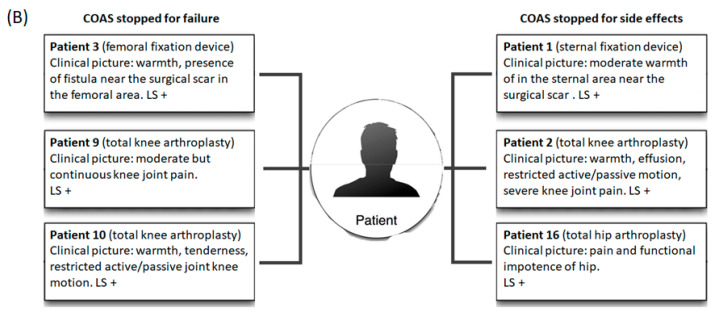
(**A**) Schematic results of COAS monitoring and follow-up; (**B**) Clinical pictures at the COAS failure. Clinic: clinical picture; CRP: C reactive protein (mg/dL); LS: Radiolabeled Leukocyte scintigraphy; COAS: chronic oral antimicrobial suppression; CDF: COAS discontinued for failure; (*): COAS discontinuation for side effects; LS+: positive radiolabeled leukocyte scintigraphy; Pos: positive; Neg: negative; ND: not done; Relapses highlighted in bold.

*Start of COAS (T1)*: All isolates were sensitive to minocycline with MIC < 0.5 mg/L. As a consequence, COAS was conducted using one oral minocycline 100 mg tablet q12h; Figure 2 summarizes the results of COAS monitoring. The overall median time of COAS follow-up available for the cohort was 15 months (min 6 months; max 30 months).

*Six-month follow-up (T2)*: As per protocol, all enrolled patients reached T2. Moreover, 2/16 (12.50%) patients presented clinical signs of infection relapse, confirmed by a new CRP elevation and positive LS scan, and were discontinued from COAS and from study. On the other hand, a further improvement of clinical condition or no relapse after the cure of the chronic infection were reported in 14/16 (87.50%) cases.

Laboratory evaluation showed that CRP was normal in 7/16 (43.75%) cases, and 9/16 (56.25%) patients had persistent positive CRP values, but in 6/9 (66.66%) cases it remained no more than two times above the normal value after a significant reduction as compared to the levels of the acute phase of infection.

LS evidenced scintigraphy signs of residual infection in 8/16 (50%) patients despite 6 of them presenting clinical and laboratory improvement.

*Twelve-month follow-up (T3)*: Overall, 14/16 (87.50%) patients reached T3. Furthermore, 7/14 (50%) subjects had both a clinical picture and normal CRP values; the other 5/14 (35.71%) presented a clinical picture not suggestive of an infection associated with abnormal CRP levels.

Additionally, 2/14 (14.28%) patients developed symptoms compatible with clinical relapse (local pain, previously not present). LS and CRP confirmed the suspicion of local infection and one of the patients was discontinued from COAS (in the second patient, COAS was previously stopped due to side effects).

LS was repeated in all six patients who at the six-month follow-up still had positive LS despite the clinical and laboratory improvement: at a median time of 13 months of COAS (min 11 months, max 14 months), the LS was negative in five of them, while it remained unchanged in one patient who underwent relapse.

*Last follow-up available (T4)*: 7/16 patients had a follow-up longer than 12 months, with a median time of COAS of 21 months (min 15 months; max 30 months). At T4, 5/7 (71.42%) patients were still taking COAS without clinical signs of the recrudescence of infection. Compared to the previous results, the T4 LS scan was confirmed negative in the five patients without clinical sign of infection. On the other hand, LS became positive in the two patients who previously stopped COAS due to side effects (although it was negative at the 6-month follow-up).

*Adverse effects*: One case of possible minocycline-induced teeth staining, and two cases of epigastric pain related to drug intake were reported. No other adverse events were observed during the COAS. No cases of *Clostridioides difficile* infection were registered.

*Adherence*: Acceptable (from 90% to 100%) long-term self-reported adherence was observed in all patients except those with an adverse event; in these three subjects the intake of the COAS was spontaneously discontinued.

*COAS discontinuation and trial of stopping*: COAS was discontinued due to the ineffectiveness of the salvage therapeutic approach in three patients and due to adverse effects of antibiotic in another three patients. Out of the three patients who had stopped the COAS due to adverse effects, one had a clinical relapse 5 months after the discontinuation of the antibiotic; while two remained in a condition of clinical quiescence without symptomatic exacerbations (but with a positive CRP) for 9 and 13 months after discontinuation, respectively, before the clinical relapse. (Figure 2)

*Overall results of COAS treatment and comparison between successful and unsuccessful treatment groups*: Overall, 10/16 (62.50%) patients were still taking COAS with no relapse at the last evaluation available. When we compared the characteristics of the patients with therapeutic success versus failure, no differences were observed between the two groups. In particular, the outcome of the treatment was not linked to sex (five (50%) females vs. two (33.3%) males, *p* = 0.63), age (74 (14.8) vs. 78.2 (13.7) years, *p* = 0.57), use of prosthesis or internal fixation device (7 (70%) vs. 4 (66.7%) prosthesis, *p* = 1), PJI instead of FRI (7 (70%) vs. 4 (66.7%) PJIs, *p* = 1).

**Figure 3:**
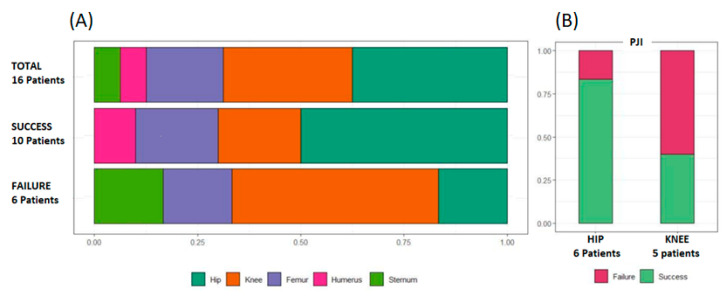
Results of COAS treatment (**A**) based on the anatomical location of the prosthesis or internal fixation device and (**B**) and in subgroup of subjects with hip of knee joint prosthesis. Legend: PJI: prosthetic joint infection.

The results of COAS treatment, based on the anatomical placement of internal fixation devices and articular prosthesis, are shown in Figure 3A. Limited to subjects with joint prosthesis infection, we observed that clinical success had a higher proportion in patients with hip arthroplasty (5/6; 83.33%) compared to those with knee arthroplasty (2/5, 40%) (Figure 3B).

## 4. Discussion

Currently, a shared and reliable strategy for patients not eligible for standard treatments of chronic-late PJI and FRI, based on the combination of surgical removal of the prosthesis or internal fixation device with antibiotic treatment, is not available. [16,17]. In this case, a debridement procedure plus a targeted antibiotic treatment followed by COAS is often adopted and can be considered as an interesting palliative approach [7,18,19]. Anyway, despite several studies demonstrating that the chronic suppression of periprosthetic joint infection with antibiotic therapy increases infection free survivorship [18,19,20], there are no large-scale validation studies of this procedure and of the methods for monitoring its performance.

In our study, we retrospectively observed a cohort of patients affected by chronic PJI and FRI who were ineligible for standard treatment and were treated using a debridement procedure with the retention of prosthesis or internal fixation device, combined with long-term antibiotic treatment and subsequent indefinite COAS. Overall, 62.50% of patients were still taking COAS with no relapse after the cure at the last evaluation available. Regarding the patients who had presented clinical failure with a relapse of the infection (37.50%), as many as 50% of them had previously stopped taking the COAS following a side effect of the antibiotic used.

Although our cohort is not large enough to draw certain conclusions, this study seems to show that COAS was effective in preventing infectious recurrence or disease progression in two-thirds of cases. Furthermore, the effect of this therapeutic strategy seems to last as long as the antibiotic intake continues; in fact, the discontinuation of COAS was always followed by therapeutic failure in our cohort. Moreover, patients’ tolerability to the COAS therapeutic strategy was burdened by adverse events (18.75%), often of modest clinical impact and consistent with the generic side effects of minocycline [21] but sufficiently disturbing to induce interruption of the chronic treatment. If these situations are excluded, self-reported adherence to treatment was acceptable, probably due to the simplicity of the therapeutic scheme.

The rate of infection recurrence and medication-related adverse events observed in our case series overlap with data reported in the literature [22,23,24,25], i.e., the survival rate without an event at 2 years was 61% in a national cross-sectional cohort study of subjects >75 years old and treated with COAS for PJI; moreover, an adverse antibiotics reaction was reported 18% of patients enrolled [21]. Similarly, 28.2% patients failed and 18% had adverse events in a cohort of subjects with PJI who were treated with tetracycline-based COAS [22].

Conceptually, the reduction in the bacterial inoculum by the debridement of infected tissues appears to be an important prerequisite for the success of COAS. Despite that, the failure of the COAS was not associated with the absence of a previous debridement in some previously published studies [15,26,27]. In any case, this procedure allows clinicians to obtain valuable samples for microbiological cultures, favoring the possibility of COAS success. Indeed, the culture of specimens from sinus tracts is not usually representative of the actual etiology.

In our cohort, all patients underwent a debridement procedure with consensual microbiological sampling, prior to the initial intensive antibiotic treatment and the onset of COAS. Per protocol, only patients with microbiological isolations of staphylococci were selected; all strains were oxacillin-resistant and sensitive to minocycline. Consequentially, all patients enrolled were treated with minocycline-based COAS.

Minocycline is a broad-spectrum second-generation tetracycline antibiotic with anti-staphylococcal activity [28,29,30]. The pharmacology of tetracycline drugs show that these molecules have a high affinity for the bone mineral matrix and are retained at high levels, also after the termination of antibiotic therapy [28]. The simplicity and acceptability of minocycline-based COAS and the pharmacoeconomic sustainability make this drug an interesting option in patients with infection due to pathogens being sensitive to minocycline and not eligible for the standard treatment of PJI and FRI. Previous studies showed that PJI managed with debridement, implant retention and extending therapy with oral antibiotics had no increase in drug-related adverse events, demonstrating safety [31]. The impact of gross side effects was also low in our cohort despite the long-term treatment carried out; in fact, despite 18.7% of subjects discontinuing COAS for possible drug-related adverse events (one case of possible minocycline-induced teeth staining and two cases of epigastric pain related to drug intake), none presented life-threatening events. On the other hand, previous studies on the long-term administration of minocycline conducted in different setting, such as the treatment of acne, showed that minocycline might increase the risk of autoimmune hepatitis, polyarteritis nodosa, and systemic lupus erythematosus, after 1 year of use. However, it should be emphasized that the population analyzed in these studies was young (unlike the population treated with COAS, which is generally elderly), and this could create a bias in the interpretation of the data [32].

Finally, non-antibiotic biological effects for minocycline were reported; the anti-inflammatory and immunomodulatory effects previously described could contribute to ameliorating the treatment outcomes and reducing the impact of local and systemic inflammation [33].

Despite the comforting available data, the need for further insights remains, in order to elucidate unclear drug retention/release mechanisms from the bone matrix and the impact of minocycline on the microbiota and the gut–bone axis.

Regarding the follow-up, the most significant difference between this study and previously published ones was the performance of serial LS. Our data showed that neither the clinic, nor the CRP nor the LS, when taken individually, seem able to predict a future COAS failure. Nevertheless, LS was able to detect the ongoing persistence or the re-activation of the infectious process more accurately than clinical evaluation and CRP. Moreover, it also helps to discriminate a mismatch between clinical and laboratory values in this setting, showing if and where a persistence of the infection-related inflammatory process is present. Therefore, LS seems to be able to provide added value to clinical monitoring, especially when the clinical and laboratory data are unable to adequately settle between the success and failure of the COAS.

However, it should be underlined that LS is able to mark active infectious processes, but it is not clear if it also adequately traces the presence of mature biofilms that are highly tolerant to host immune defenses [34,35]. Therefore, its negativity does not exclude the presence of a viable biofilm potentially able to reactivate/exacerbate a local infectious process.

This aspect was particularly important in our setting where the microbiological isolates were limited to *Staphylococcus aureus* and coagulase-negative staphylococci, i.e., a group of potentially biofilm-producing bacteria. Gram-positive bacteria, such as *Staphylococci*, are typical causative pathogens of these infections, often characterized by high resistance levels to the host’s immune response and antibiotic therapy [36,37]. In fact, these microorganisms may persist in biofilm-based colonies or be intracellular, hidden within osteoblasts. These evasion strategies to the immune control may also contribute to reducing the efficacy of antibiotics in targeting these pathogens but also the ability of diagnostic tests to monitor the infection [38,39,40].

Of particular interest is that in previous studies minocycline showed a concentration-dependent activity against biofilm-embedded MRSA [41,42]. Similarly, minocycline also demonstrated high in vitro activity for preventing biofilm formation against other microorganisms such as *Acinetobacter baumannii* [43]. The high affinity of tetracyclines for the bone mineral matrix, which are retained at high levels even after treatment interruption, could represent an element favoring the anti-biofilm activity of the minocycline in this setting [30].

This study has several limitations and requires a prudential approach to the results. First, the retrospective nature of the research reduced the opportunity to investigate in more depth. The number of patients enrolled was small, but the poor frequency of these conditions still makes the analysis of the sample interesting, especially when it has been monitored for a long period. Despite this, it is important to point out that the heterogeneity of the concepts of “healing”, “cure” and “failure” in the literature is a possible limit in the comparative evaluation between the results of the different studies on the topic.

The data presented are limited to patients without a fistula/draining sinus at baseline. Indeed, no patient in our cohort had this condition at baseline. This is of relevance, considering that COAS appears to have limited utility in the presence of a sinus [44].

Moreover, patients enrolled underwent LS without the withdrawal of antibiotic therapy, and it is not yet fully clarified whether this condition could influence the results of the examination [45]. Finally, although scintigraphy is considered a useful diagnostic tool for PJI and FRI, there are still no studies about its role in COAS treatment monitoring; therefore, it is not possible to compare our results and further studies with a larger sample size are needed to better understand the topic.

## 5. Conclusions

COAS can be an interesting strategy for infection control in more than a half of patients affected by Gram-positive chronic PJI and FRI and is not suitable for standard eradication treatment. However, the significant risk of clinical relapse and side effects related to the treatment must also be taken into due consideration when choosing this strategy. The topic of monitoring COAS’ effectiveness, which has not yet been defined, is relevant. In this sense, this is the first study to provide insight into the potential role of scintigraphy as a tool for diagnostic support and follow-up in patients treated with COAS. Standard tools of follow-up still need to be validated, but in this setting a combination of clinical, laboratory and LS evaluation seems to monitor the infection properly. Finally, we underlined that minocycline-based COAS can be a useful, safe, and cost-effective targeted option in patients with microbiological isolates sensitive to minocycline.

## Figures and Tables

**Table 1:** Characteristics of patients enrolled at baseline (T0). CONS: coagulase-negative *Staphylococci*; CRP: C-reactive protein (mg/dL); FRI: fracture-related infection; MRSA: Methicillin-Resistant *Staphylococcus aureus*; PJI: periprosthetic joint infection.

Demographic	Age (±SD)	75 (±14)
	Sex F/M	9 (56%)/7 (44%)
Clinical and laboratory	Overall PJI	11 (69%)
	Hip	6 (54.55%)
	Knee	5 (45.45%)
	Overall FRI	5 (31%)
	Femur	3 (60%)
	Sternum	1 (20%)
	Humerus	1 (20%)
	CRP at presentation	4.5 (±5.70)
Microbiology	*CONS*	13 (82%)
	*CONS + E. coli*	1 (6%)
	*MRSA*	2 (12%)

## Data Availability

The data presented in this study are available on request from the corresponding author.
